# Development of Chemical Tools for the Human YEATS Domain

**DOI:** 10.1021/acschembio.5c00349

**Published:** 2025-07-11

**Authors:** Xuejiao Shirley Guo, Abdalrahman Khalifa, Karthik Selvam, Tatiana G. Kutateladze, Wenshe Ray Liu

**Affiliations:** † Texas A&M Drug Discovery Center and Department of Chemistry, 14736 Texas A&M University, College Station, Texas 77843, United States; ‡ Department of Pharmacology, 12225 University of Colorado School of Medicine, Aurora, Colorado 80045, United States; § Institute of Biosciences and Technology and Department of Translational Medical Sciences, College of Medicine, Texas A&M University, Houston, Texas 77030, United States; ∥ Department of Biochemistry and Biophysics, Texas A&M University, College Station, Texas 77843, United States; ⊥ Department of Cell Biology and Genetics, College of Medicine, Texas A&M University, College Station, Texas 77843, United States; # Department of Pharmaceutical Sciences, Texas A&M University, College Station, Texas 77843, United States

## Abstract

The YEATS domain is an evolutionarily conserved epigenetic reader that specifically recognizes post-translational lysine acylation on histone tails and plays a crucial role in chromatin remodeling and transcriptional regulation. Four human YEATS domain-containing proteins, ENL, AF9, YEATS2, and GAS41, have been implicated in the pathogenesis of various malignancies. This review provides an overview of the structural basis for the YEATS domain’s recognition of diverse acyllysine post-translational modifications and discusses the disease-related consequences of aberrant YEATS activity. Emphasis is placed on recent progress in the development of chemical modulators, including peptide-based inhibitors, small molecules, and proteolysis-targeting chimeras (PROTACs), that represent promising strategies for the selective targeting of YEATS domains. These developments underscore the potential of YEATS-directed therapies in epigenetic drug discovery.

## Graphical abstract

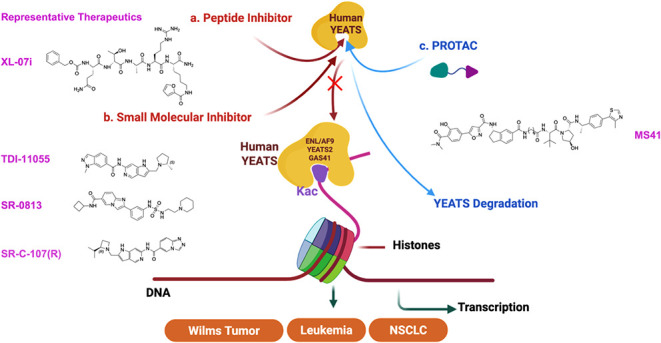

## Introduction

The fundamental unit of chromatin, the nucleosome, consists of an octamer of histone proteins H2A, H2B, H3, and H4, around which DNA is wrapped almost twice. Histones, particularly their N-terminal and C-terminal tails, undergo numerous post-translational modifications (PTMs). These PTMs, often referred to as epigenetic marks, mediate gene transcription, DNA repair, cell growth, and differentiation.^1–4^ Among the most common histone PTMs are methylation, acetylation, phosphorylation, and ubiquitination, with each playing a distinct role in biological processes.^5,6^ Lysine acetylation was the first modification discovered and characterized by Allfrey et al. in 1964.^7^ Acetylation neutralizes the positive charge of the lysine side chain and usually leads to a more open chromatin state and active gene transcription. This modification is installed by histone acetyltransferases (HATs), named “writers”, and is removed by histone deacetylases (HDACs), named “erasers”. Twenty-one HATs and 18 HDACs, identified in the human proteome, form diverse families of enzymes based on their substrate selectivities and catalytic mechanisms.^8,9^

Lysine acetylation (acetyllysine, Kac), or generally lysine acylation (acyllysine, Kacyl), is recognized by “reader” domains, such as bromodomains, double plant homeodomain fingers (DPFs), and the Yaf9-ENL-AF9-Taf14-Sas5 (YEATS) domain.^5,6,10^ In this work, we review the molecular mechanisms by which human YEATS readers interact with acyllysine PTMs and highlight the significance of these interactions in normal biological processes and disease. We also summarize recent advances in the development of YEATS-specific chemical tools to facilitate discoveries of novel drug design approaches.

## The YEATS Domain is an Acyllysine Reader

The YEATS domain is present in 134 proteins spanning 59 species, which underscores its evolutionarily conserved role in chromatin biology. There are three YEATS domain-containing proteins in , Yaf9, Sas5, and Taf14, one in , D12, and four in mammals, ENL (MLLT1/YEATS1), AF9 (MLLT3/YEATS3), GAS41 (YEATS4), and YEATS2 (Figure 1). Early insights into the YEATS domain function emerged in 2005: ENL was shown to colocalize with leukemogenic fusions in nuclear speckles and directly interact with histones H3 and H1 in a YEATS-dependent manner.^11^ A breakthrough followed in 2009, when the crystal structure of the yeast Yaf9 YEATS domain was determined by the Kobor group.^12^ The structure revealed an immunoglobulin (Ig)-like β-sandwich fold of the YEATS domain consisting of eight antiparallel β-strands and capped with two short α-helices.^12^ A deep, narrow pocket was also mapped and proposed to serve as a histone ligand-binding site. Five years later, in 2014, the Shi and Li groups identified and characterized the YEATS domain of human AF9 as a selective reader of acetylated lysine 9 of histone H3 (H3K9ac) (Figure 2).^13^

**Figure 1:**
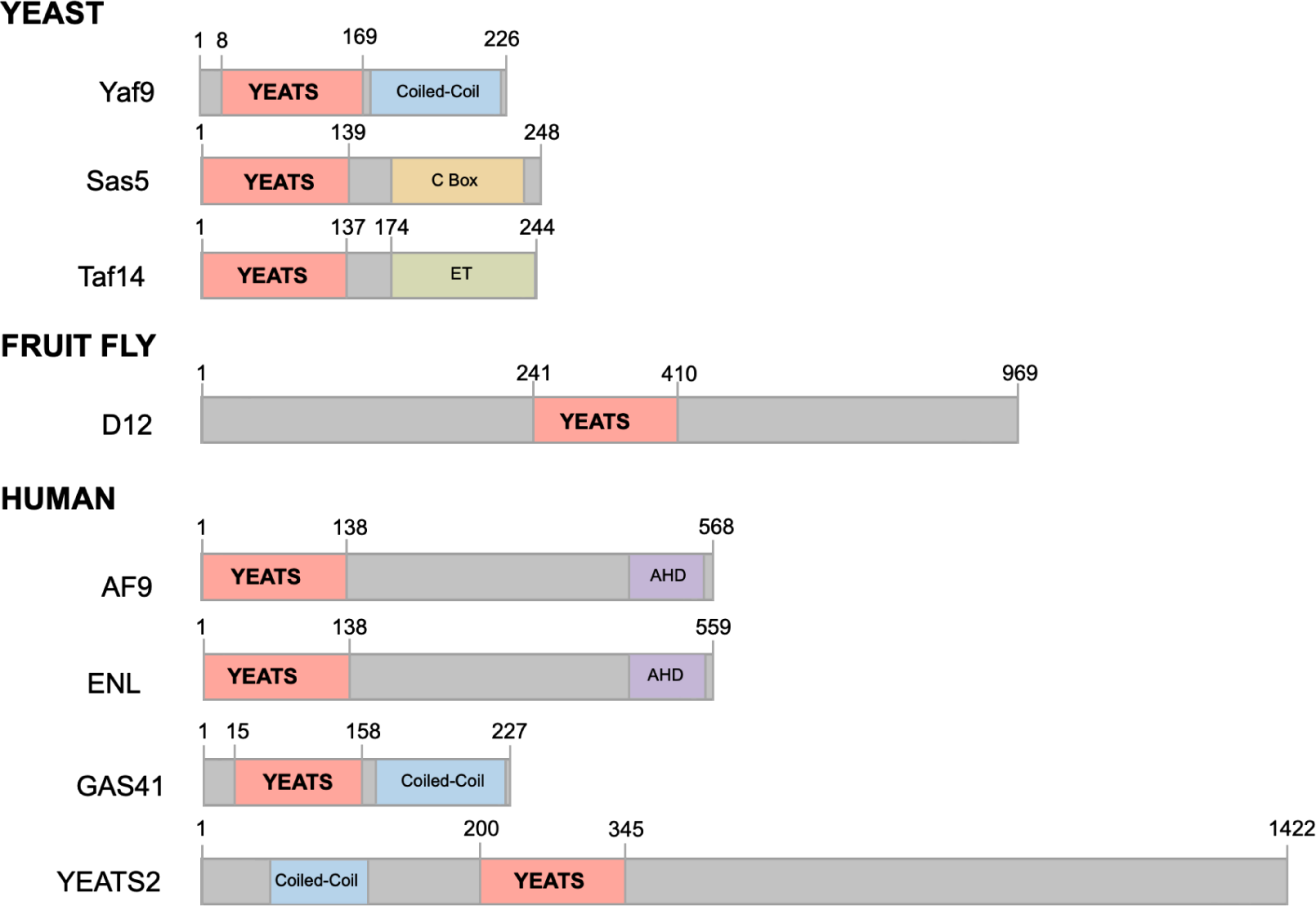
Domain architecture of YEATS-containing proteins. ET, extraterminal; AHD, ANC1 homology domain.

**Figure 2:**
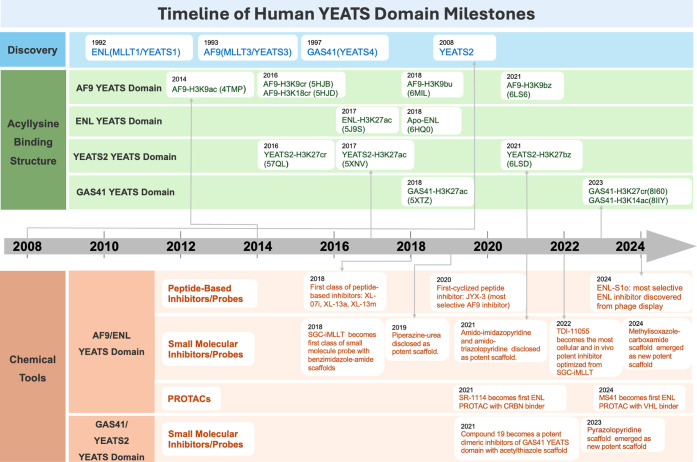
Timeline of the human YEATS domain studies. The discovery of four human YEATS domain-containing proteins, structures of the YEATS domain complexes with PDB codes, and representative chemical probes are chronologically listed.

## The Acyllysine-Binding Mechanism of the YEATS Domain

The crystal structure of the AF9 YEATS domain in complex with the H3K9ac peptide revealed a unique π–π stacking binding mechanism, distinct from the mechanisms described previously for bromodomains or DPFs.^13^ The H3K9ac-binding pocket of the AF9 YEATS domain contains five aromatic residues, F28, H56, F59, Y78, and F81, and serine S58 that are involved in hydrophobic, π–π stacking, and polar interactions with acetyllysine (Figure 3). The acetyl group of K9ac is positioned between F59 and Y78, whereas the side chain amide of K9ac is restrained by hydrogen bonds formed with the hydroxyl group of S58 and the backbone amide of Y78 and through a water-mediated hydrogen bond with A79. Residues flanking K9ac also contribute to the binding to AF9-H3R8 forms hydrogen bonds with D103; H3T6 occupies a shallow pocket created by A79, L106, and L108; H3Q5 interacts with R8 and D103; and the side chain of H3K4 is involved in hydrophobic contacts with H107 and H111.

**Figure 3:**
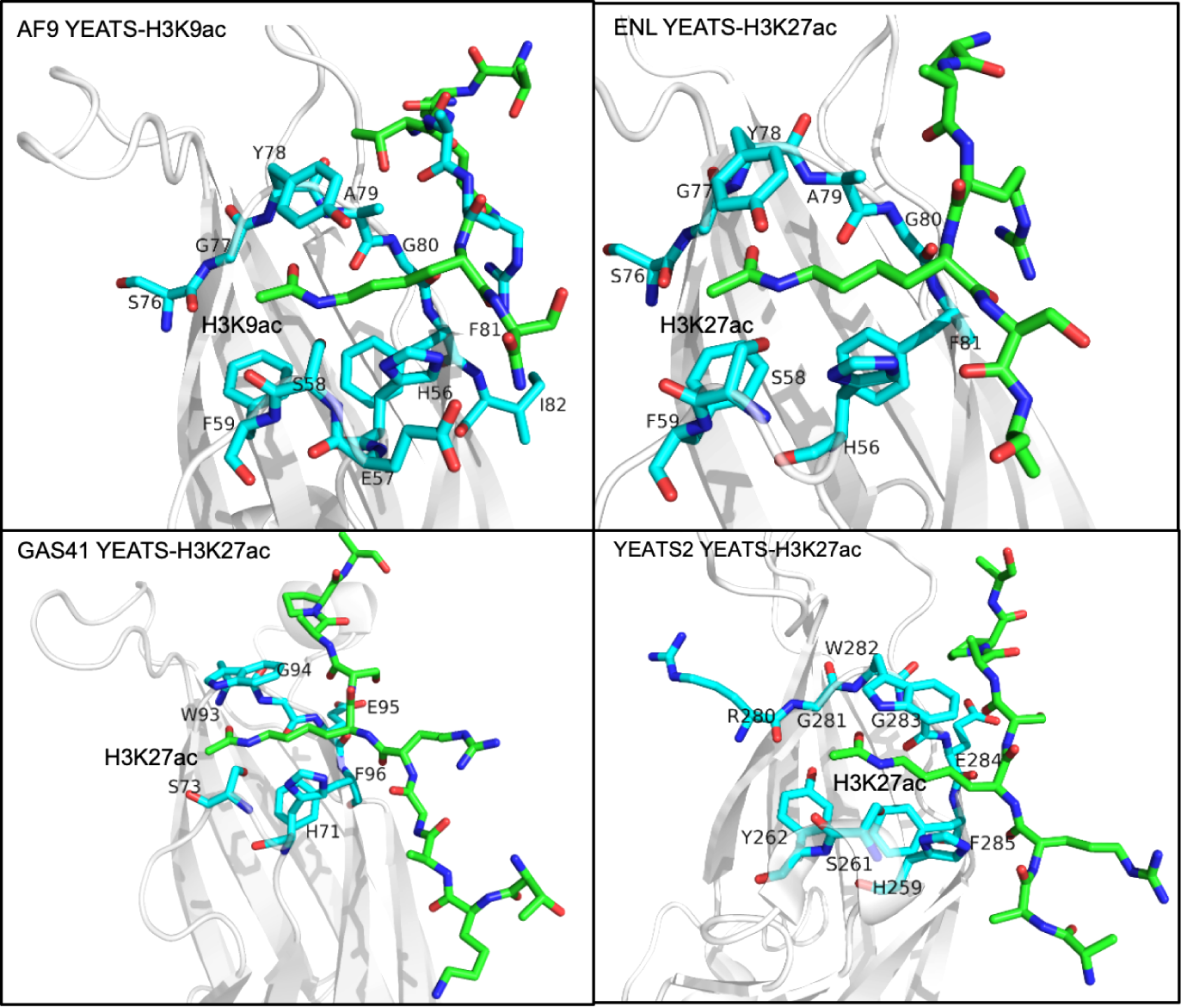
Structural comparison of the acetyllysine-binding sites of the YEATS domain family.Close view of the crystal structures of the YEATS domains from AF9 (PDB: 4TMP), ENL (PDB: 5J9S), YEATS2 (PDB: 5XNV), and GAS41 (PDB: 5XTZ) in complex with indicated acetylated histone peptides (green). Acetyllysine-binding site residues of the YEATS domains are shown as sticks, labeled, and colored cyan. The structures demonstrate a conserved acetyllysine-binding mode across the YEATS family members.

The AF9 YEATS domain binds to H3K9ac with a dissociation constant (*K*_d_) of 3.7 μM but is also capable of recognizing H3K18ac and H3K27ac, albeit ∼3–4 times weaker, and shows no interaction with H3K14ac or acetylated H4. Unlike the amino acid sequence of H3K14ac, the H3K9ac, H3K18ac, and H3K27ac sequences contain an arginine in the position prior to acyllysine and a small hydrophobic residue (sm) in the position prior to this arginine. The presence of the sm-RKac motif in H3K18ac and H3K27ac may explain the ability of the AF9 YEATS domain to associate with these PTMs, although with a slightly reduced affinity. In addition to recognizing acetylation marks, the AF9 YEATS domain can also bind other acylation modifications, including crotonylation (Kcr), propionylation (Kpr), and butyrylation (Kbu), but not bulkier modifications, such as succinylation (Ksu) or 2-hydroxyisobutyrylation (Khib).^14,15^ Crotonylation enhances binding of the AF9 YEATS domain 2-fold,^15^ and a noncanonical π–π–π stacking mechanism originally identified in the yeast counterpart Taf14 ^10^ may play a role.

Subsequent studies revealed that the overall acyllysine-binding mechanism is conserved in YEATS domains from other human proteins, such as ENL, YEATS2, and GAS41, but several structural and mechanistic dissimilarities were also observed. For example, the ENL YEATS domain has a stronger preference for Kcr over Kac.^16^ The YEATS2 YEATS domain exhibits weak binding to H3K27ac and does not recognize other acetylated sites.^17^ The acetyllysine-binding site (loops L3, L5, and L7) in the YEATS domain of YEATS2 is less negatively charged than that in AF9, likely accounting for the reduced binding affinity. A unique S230 residue of the YEATS2 YEATS domain allows for the recognition of Khib, which may be essential for active transcription in germ cells.^18^ Unlike the YEATS domains of AF9 and ENL, which preferentially bind H3K9ac and H3K27ac, the GAS41 YEATS domain selects for H3K27ac and H3K14ac, and this difference in binding selectivity suggests a distinct functional role of GAS41. The H3K27ac-binding mode of the YEATS domain of GAS41 is similar to the binding mode of the YEATS2 YEATS domain, in which the Kac-X-X-Pro motif of histone H3 is bound in a relatively flat hydrophobic pocket of the domain.^17,19^ More recently, the Umehara group identified a unique pocket located far from the Kac-binding site in the structure of the MBP-GAS41 YEATS-H3K14ac complex.^20^ This pocket accommodates the N-terminus of H3 (H3NT), which stabilizes the complex and enhances the binding affinity.

## Role of the Human YEATS Family in Disease

Four human YEATS family proteins are core components of chromatin-associated complexes that play pivotal roles in many fundamental DNA-templated processes, including transcription and chromatin remodeling. Aberrant functions of the YEATS proteins are associated with various diseases, particularly cancer. Both ENL and AF9 are coregulators of the Super Elongation Complex (SEC), which also contains the transcription elongation factors CDK9, ELL2, and other coregulators such as AFF4 and AFF1.^15^ While the C-terminal regions of ENL and AF9 interact with AFF4, the distinctive acyllysine-binding activities of their YEATS domains (ENL prefers H3K27acyl, whereas AF9 prefers H3K9acyl and H3K18acyl) can provide selectivity for the recruitment or stabilization of SEC at specific genomic loci. In aggressive MLL fusion-driven leukemias AML and ALL, oncogenic fusion proteins, e.g., MLL-AF9, MLL-ENL, recruit SEC to MLL target loci, overly activating the *HOXA* gene family, a well-established hallmark of leukemia.^21,22^

ENL itself displays oncogenic properties.^16^ Genomic analysis shows that ENL associates with a broader set of genes compared with MLL-fusion proteins in leukemia cell lines MOLM-13 and MV4-11, suggesting a more widespread role in transcriptional regulation. The genes bound by ENL in leukemia are enriched in pathways implicated in blood cancer and hematologic diseases. ENL-binding sites near transcription start sites are also enriched in H3K9ac and H3K27ac and show a high degree of co-occupancy with promoter-proximal RNA Pol II. Depletion of ENL or mutations in the YEATS domain result in a reduction of Pol II and CDK9 levels at these sites, underscoring the role of the YEATS domain in the recruitment of Pol II and SEC. Furthermore, a genome-scale loss-of-function screen in an MLL-AF4-positive acute leukemia cell line identified ENL as a critical gene for leukemic cell proliferation both in vitro and in vivo.^16,23^ Acute ENL depletion impairs both the initiation and elongation of Pol II at active genes across the genome, with pronounced effects at genes showing high ENL occupancy.^23^ Unlike ENL, AF9 is not required for leukemia maintenance, indicating that these proteins have distinct roles in transcriptional regulation.^16^ In addition to supporting the growth of leukemic cells, the ENL YEATS domain plays a critical role in MLL-ENL-driven leukemogenesis. The Muntean group reported that the ENL YEATS domain is retained in approximately 84% of patients harboring MLL-ENL fusions.^24^ The deletion of the YEATS domain considerably decreased the level of binding of the MLL-ENL fusion protein to chromatin and led to a reduced level of expression of key leukemogenic genes, including *Eya1* and *Meis1*, in the mouse model.

Recent studies have identified recurrent hotspot mutations, including small insertions and deletions, in the YEATS domain of ENL in Wilms tumor, the most prevalent pediatric renal malignancy. Among them, p.117_118insNHL (T1), p.112_114PPV > L (T2), and p.111_113NPP > K (T3) mutations have been the most studied due to their high frequencies.^25^ The Wang group found that these mutations impart ENL gain-of-function characteristics, promoting aberrant transcriptional activation through the formation of biomolecular condensates at specific genomic loci. When introduced into mouse embryonic stem cells, the mutant ENL induced Wilms tumor-like blastemal structures under differentiation conditions, corroborating its role in tumorigenesis.^26,27^ Furthermore, these mutations disrupted normal kidney development by altering cell composition, lineage specification, and transcriptional networks and ultimately led to defective nephrogenesis and postnatal lethality in mouse models,^28^ whereas inhibition of the acyllysine-binding activity of ENL restored gene expression profiles and developmental progression in embryonic kidney tissues in vivo.^29^ ENL overexpression also promotes colorectal cancer tumorigenesis in both in vitro and in vivo models, and targeted degradation or inhibition of ENL, when combined with BET inhibitors, shows synergistic effects in suppressing colorectal cancer growth.^30^

A well-characterized function of ENL and AF9 is the association with the H3K79-specific disruptor of telomeric silencing 1-like (DOT1L) methyltransferase and the formation of a DOT1L-containing complex (DotCom) that produces H3K79me2, a modification associated with actively transcribed genes.^31,32^ AF9 recruits DOT1L to active gene loci in a YEATS-dependent manner, facilitating H3K79 dimethylation and promoting transcriptional activation,^13^ and ENL depletion results in a reduced level of DOT1L-mediated H3K79 dimethylation at target genes.^16^ Recent genomic and mechanistic studies have shown that ENL and MOZ (monocytic leukemia zinc finger) co-occupy active promoters, with MOZ guiding ENL recruitment.^33^ ENL assembles at the intrinsically disordered region (IDR) of MOZ through both acetylation-dependent and -independent mechanisms: the YEATS domain of ENL binds MOZ acetylation sites, allowing for the recruitment of transcriptional cofactors via the ENL ET domain, while ENL ET-mediated acetylation-independent binding to MOZ facilitates chromatin association through the recognition of H3K27 and H3K18 acylation marks by the YEATS domain.

YEATS2 is a core subunit of the histone acetyltransferase Ada-two-A-containing (ATAC) complex that generates H3K9ac and H3K14ac and is essential in chromatin regulation and transcriptional activation in metazoans. YEATS2 facilitates ATAC recruitment to transcriptionally active regions to maintain an open chromatin state through binding to H3K27ac by its YEATS domain.^34,35^ YEATS2 is frequently upregulated in human cancers, including lung squamous cell carcinoma (56%), ovarian serous cystadenocarcinoma (27%), and head and neck squamous cell carcinoma (23%), with its expression strongly correlating with gene amplification and poor clinical prognosis. In nonsmall cell lung cancer (NSCLC) cell lines, such as H1299, coexpression of YEATS2 with GCN5 or PCAF enhances global histone acetylation, especially at H3K9. YEATS2 knockdown reduces cell viability, growth, and transformation capacity, pointing to its role in promoting tumor cell proliferation.^17^

Amplification of GAS41 is also frequently observed in NSCLC, and GAS41 knockdown has been shown to suppress NSCLC growth. GAS41 is a component of the Tip60-p400 and SRCAP chromatin remodeling complexes, both of which are involved in mediating the incorporation of the histone variant H2A.Z in place of canonical H2A. RNA-seq analysis following GAS41 knockdown in H1299 cells shows significant downregulation of genes associated with the cell cycle and DNA replication, many of which are normally characterized by H2A.Z. GAS41 depletion decreases total H2A.Z levels, despite unchanged expression of H2A.Z-encoding genes, but restoration of H2A.Z levels does not rescue defects in chromatin loading, gene expression, or cell proliferation, revealing a critical role of GAS41 in enabling H2A.Z deposition at loci necessary for NSCLC cell survival.^36^ The defects caused by GAS41 knockdown, including reduced H2A.Z protein levels and impaired cell growth, were reversed by the reintroduction of wild-type GAS41 but not by the reintroduction of the mutants deficient in acyllysine binding. In support, chromatin immunoprecipitation sequencing (ChIP-seq) showed that GAS41 colocalizes with H3K27ac and H3K14ac, the histone acetylation marks recognized by its YEATS domain.^37^ When associated with the SIN3A-HDAC1 corepressor complex, the YEATS domain of GAS41 selectively binds to H3K27cr, which leads to MYC-dependent transcriptional repression of key genes, including the cyclin-dependent kinase inhibitor p21. Loss of GAS41 or disruption of its ability to bind H3K27cr results in p21 derepression, induction of cell-cycle arrest, and suppression of colorectal tumor growth in vivo.^38^

## Chemical Tools for Targeting Human YEATS Domains

The strong association of the YEATS domain-containing proteins with diseases implies that this epigenetic reader represents a promising therapeutic target.^39^ Various chemical tools, including small-molecule, peptide-based inhibitors and probes, as well as PROTACs, have been designed and tested.^40–42^ Progresses are summarized as follows.

## ENL and AF9 YEATS Domain

### Peptide

Motivated by the unique π–π–π stacking mechanisms observed in crystal structures of the AF9 and ENL YEATS complexes with crotonyllysine peptides, Li’s group developed the first-in-class peptide-based YEATS domain inhibitors.^43^ Structure-based optimization produced selective and potent inhibitors with π-system-containing modifications for AF9 and ENL YEATS domains (Figure 4).^43^ A pentapeptide Cbz-Q-T-A-R-Kfu (XL-07i) showed 3-fold selectivity toward AF9 YEATS (IC_50_ = 0.26 μM, *K*_d_ = 0.33 μM) over ENL YEATS (IC_50_ = 1.30 μM). Replacing the 2-furancarbonyl group in XL-07i with a 5-oxazolecarbonyl group yielded another inhibitor, XL-13a, with slightly enhanced AF9 (IC_50_ = 0.24 μM, *K*_d_ = 0.13 μM) and ENL (IC_50_ = 0.71 μM) inhibition potency. In the crystal structure of the XL-07i-bound AF9 YEATS domain, the planar 2-furancarbonyl group inserts into the Kac/Kcr-binding aromatic cage, aligning parallel to the aromatic rings of F59 and Y78 and making π–π–π stacking contacts, whereas an intramolecular hydrogen bond between the side chain of the Q and R residues in XL-07i restrains its conformation (Figure 5). Additionally, the furan oxygen forms a hydrogen bond with the side-chain hydroxyl group of S58, mimicking the conserved hydrogen bond seen in the case of crotonyllysine binding. The Cbz group occupies a shallow groove outside the Kac-binding pocket and is stabilized by π-stacking with H107 and H111. In contrast, ENL contains nonaromatic Asn in place of these two histidine residues, and the binding groove in ENL appears to be too rigid to accommodate the Cbz group, likely accounting for XL-07i’s selectivity toward AF9 over ENL. Because the ENL YEATS domain selects for H3K27cr, Li and coworkers designed the tripeptide Ac-A-R-Koxa (5-oxazolecarbonyl) (XL-13m), incorporating amino acids adjacent to the K27 site and a 5-oxazolecarbonyl π-system on the lysine. XL-13m displays 5-fold selectivity for ENL YEATS (IC_50_ = 0.56 μM) over AF9 YEATS (IC_50_ = 2.5 μM). In acute leukemia cells, XL-13m reduced ENL chromatin occupancy and downregulated critical leukemogenic genes including HOXA10, MYB, MYC, and MEIS1.

**Figure 4:**
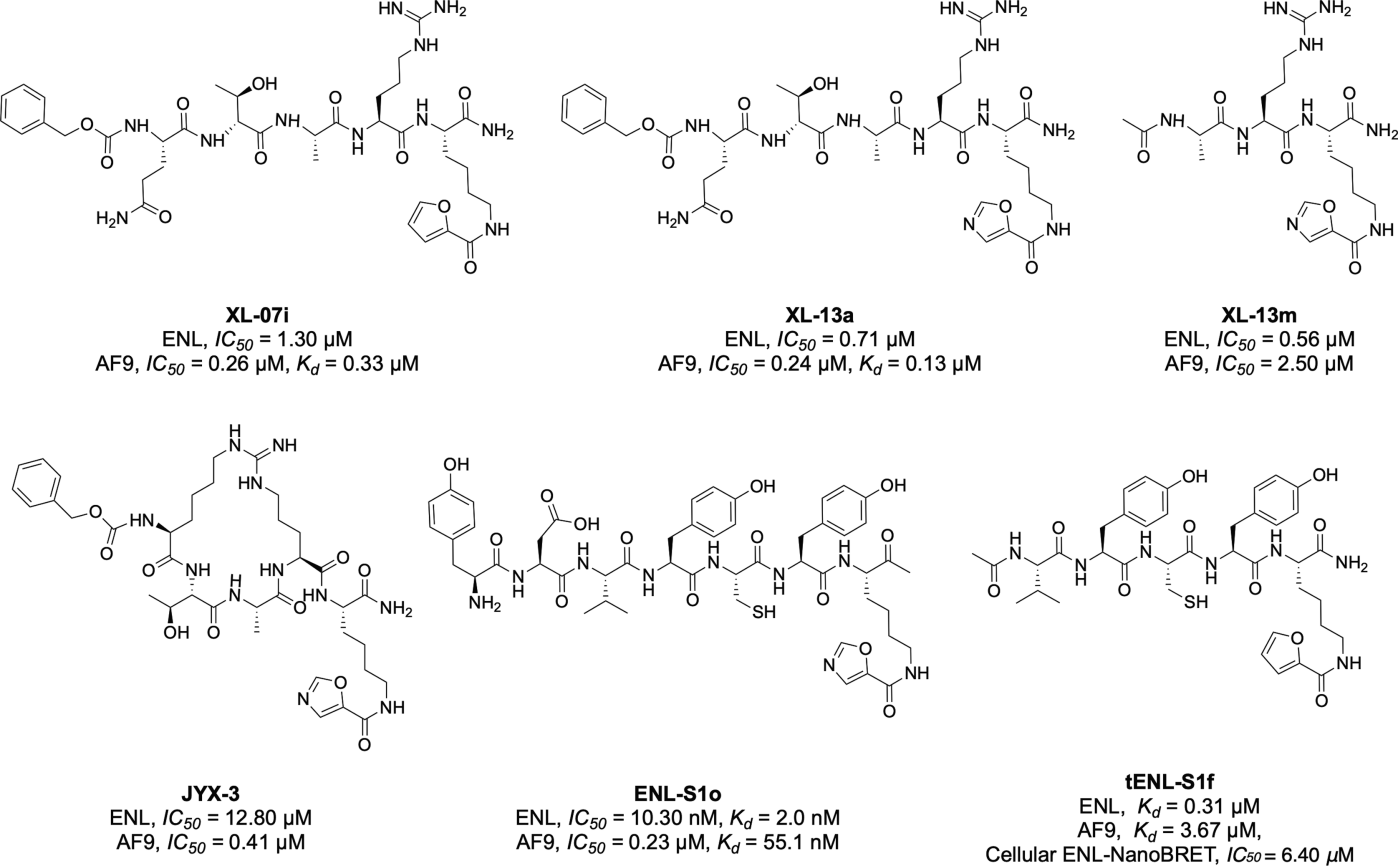
Representative peptide-based inhibitors and probes for the YEATS domains of ENL and AF9.

**Figure 5:**
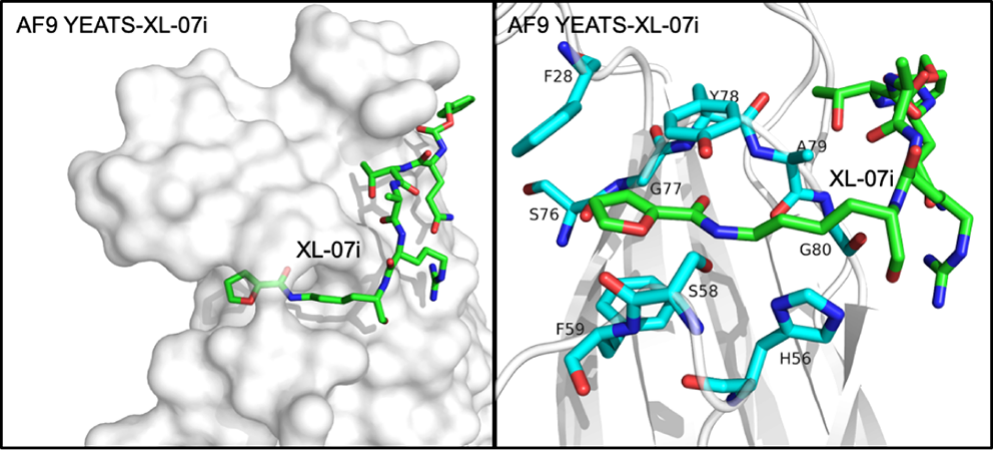
Structure of the YEATS domain of AF9 in complex with the synthetic peptide XL-07i (PDB: 5YYF). Left: Surface representation of the AF9 YEATS domain with XL-07i (green sticks) bound in the open-ended pocket. Right: A zoomed-in view of the binding pocket. The XL-07i-binding site residues of the AF9 YEATS domain are shown as sticks and colored cyan. The peptide adopts a conformation that allows its acetyllysine mimic to fully occupy the binding pocket, closely recapitulating the geometry of bound acetylated histone peptides.

However, as the ligand-binding residues within the reader pockets of AF9 and ENL are identical, it is not feasible to design inhibitors selective for only AF9 or ENL via targeting the pocket alone. Achieving selectivity requires additional interactions that the regions surrounding the reader pocket could provide. One such strategy is to cyclize linear peptides that would adopt an AF9-favored or ENL-favored conformation. This strategy was successfully implicated to generate JYX-3 that was synthesized by covalently linking the side chains of glutamine and arginine residues with a linker.^44^ JYX-3 exhibits remarkable selectivity, showing a 38-fold higher affinity for AF9 YEATS (IC_50_ = 0.41 μM) over ENL YEATS (IC_50_ = 12.80 μM), making it the most selective AF9 YEATS inhibitor reported to date. Cyclization also improved the cell permeability and protease resistance of the inhibitor.^45^ In cellular models, JYX-3 effectively reduced AF9 chromatin occupancy and downregulated transcription of its target genes.

Recently, our group developed a strategy aiming at rapid ENL inhibitor discovery using phage display combined with a genetically encoded noncanonical amino acid (ncAA) bearing an epigenetic mark.^46^ We were motivated by electrostatically tunable amide-π interactions between AF9 and acylated lysines, along with the evidence that CH-π interactions involving Phe28 are critical for acyllysine recognition.^47,48^ We guided the library toward the active site to enrich for high-affinity ligands by incorporating Nε-butyryl-l-lysine (BuK), which enhances binding via the CH-π interaction with F28 of the ENL YEATS domain. One identified hit, ENL-S1, was further optimized by replacing BuK with π-system-containing pharmacophores to exploit a unique π–π–π stacking interaction. This resulted in ENL-S1o, a selective ENL YEATS inhibitor with a *K*_d_ of 2.0 nM and 28-fold selectivity over AF9 YEATS. To enhance cell permeability and improve proteolytic stability, we removed the first two amino acids in ENL-S1o but retained the core-binding motif. The resulting analogue, tENL-S1f, showed robust cellular target engagement, effectively inhibited leukemia cell growth, and downregulated ENL target gene expression.

### Small Molecule

While peptide-based inhibitors have successfully leveraged the π–π–π stacking and hydrogen bonding contacts within the YEATS domain to achieve selective engagement, their therapeutic potential remains limited due to poor pharmacokinetics and cell permeability. To overcome these limitations and expand the chemical space of YEATS domain inhibitors, studies have shifted toward focusing on small-molecule approaches. These efforts aim to mimic key interactions observed in peptide-based inhibitors while offering improved drug-like properties, including enhanced metabolic stability, membrane permeability, and oral bioavailability. Again, as in the case of peptide-based inhibitors, due to the high sequence similarity between the YEATS domains of ENL and AF9, achieving selective inhibition has proven to be challenging. Early work aimed at developing dual inhibitors or probes that target both proteins to address potential functional redundancy. However, more recent structural studies revealed another binding pocket in both domains, a narrow, open-ended cavity, which guided a fragment-based design strategy for each domain individually. Linear molecules with central carbonyl groups as core scaffolds to mimic acetyllysine interactions have also been explored.

Two key requirements for suitable ligands were proposed by Heidenreich et al.: a central amide to mimic acetyllysine and an aromatic group at either the carbonyl (R1) or nitrogen (R2) end to engage in π-stacking with F28 and F59.^49^ On the basis of this assumption, an acyllysine mimetic molecule with a benzimidazole amide backbone, compound 20, was designed and showed strong binding to ENL YEATS as measured by ITC (*K*_d_ of 807 nM) (Figure 6). The crystal structure of the ENL-compound 20 complex (PDB ID: 6HPW) confirmed this design strategy. The amide core maintained flipped binding, making key hydrogen bonds with Y78 and S58, while the benzimidazole group enabled a π-stacking interaction with F28 and F59. Additional interactions, including hydrogen bonds involving the piperidine and benzimidazole nitrogens, further stabilized the complex.^49^ Likewise, another benzimidazole-amide compound, XS018661, identified through a peptide displacement assay, exhibited strong binding to both ENL and AF9 (IC_50_ = 1.6 and 3.0 μM; *K*_d_ = 754 and 523 nM, respectively), supporting its potential as a lead scaffold.^50^

**Figure 6:**
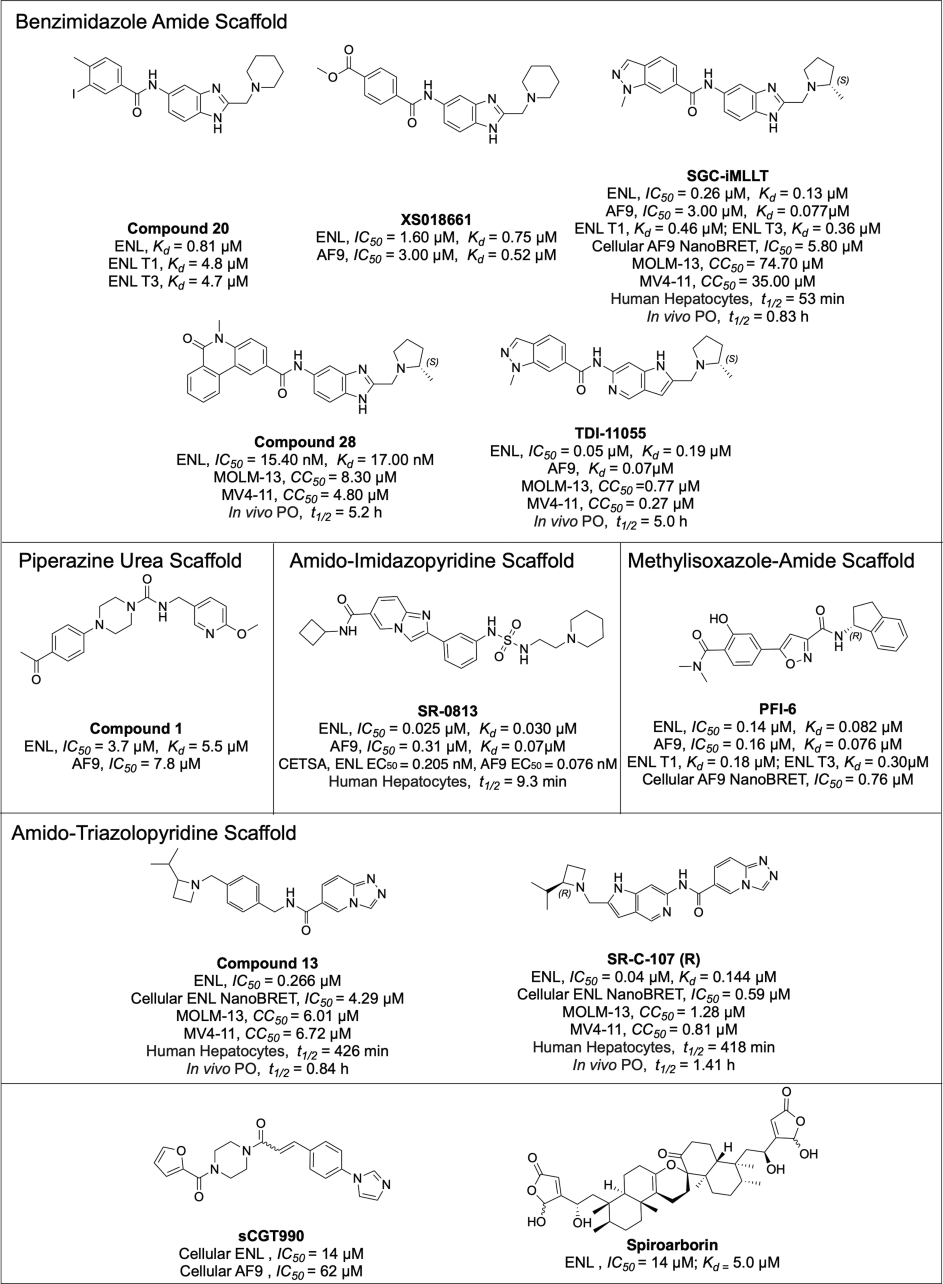
Representative small-molecule inhibitors and probes for the YEATS domains of ENL and AF9.

Building on these early hits, SGC-iMLLT developed by Moustakim et al. became the first chemical probe for YEATS domain proteins.^51^ Optimized using a structure–activity relationship (SAR) approach from an amido-benzimidazole hit, SGC-iMLLT bound ENL and AF9 with high affinity (ENL: IC_50_ = 0.26 μM, *K*_d_ = 0.129 μM; AF9: *K*_d_ = 0.077 μM) and showed remarkable selectivity over YEATS2 and GAS41 (IC_50_ > 10 μM). The crystal structure of the SGC-iMLLT-bound ENL YEATS domain (PDB ID: 6HT1) (Figure 7) revealed that the inhibitor occupies the acetyllysine-binding pocket, and the central amide forms hydrogen bonds analogously to acetyllysine. The benzimidazole moiety is engaged in π-stacking near the channel exit, while the indazole ring mimics the lysine hydrocarbon chain and is involved in CH-π interactions. Stabilizing electrostatic interactions were also observed between the pyrrolidine ring and E75.^51^ Despite its potency, SGC-iMLLT demonstrated only moderate metabolic stability (*t*_1/2_ = 53 min in primary human hepatocytes), with N-demethylation being a primary destabilizing metabolic pathway. To improve both activity and pharmacokinetics, several analogues were synthesized. Among them, compound 28, containing a methyl phenanthridinone in place of indazole, showed significantly enhanced cellular activity against MV4-11 and MOLM-13 (IC_50_= 4.8 and 8.3 μM, respectively), implying that this compound is ∼7- to 9-fold more potent than SGC-iMLLT in destroying tumor cells in vitro. Compound 28 has also improved PK properties (*t*_1/2_ = 5.2 h), demonstrating its therapeutic promise.^52^ Nonetheless, SGC-iMLLT’s limited in vivo efficacy due to poor pharmacokinetics (*t*_1/2_ = 0.83 h at 100 mg/kg PO in mice) spurred further optimization with the goal to enhance drug-like properties while preserving potency and selectivity. These efforts led to the development of TDI-11055 with high potency and selectivity for ENL (IC_50_ = 0.05 μM, *K*_d_ = 190 nM) and AF9 (IC_50_ = 0.07 μM) but negligible activity against GAS41 and YEATS2 (IC_50_ > 100 μM).^53^

**Figure 7:**
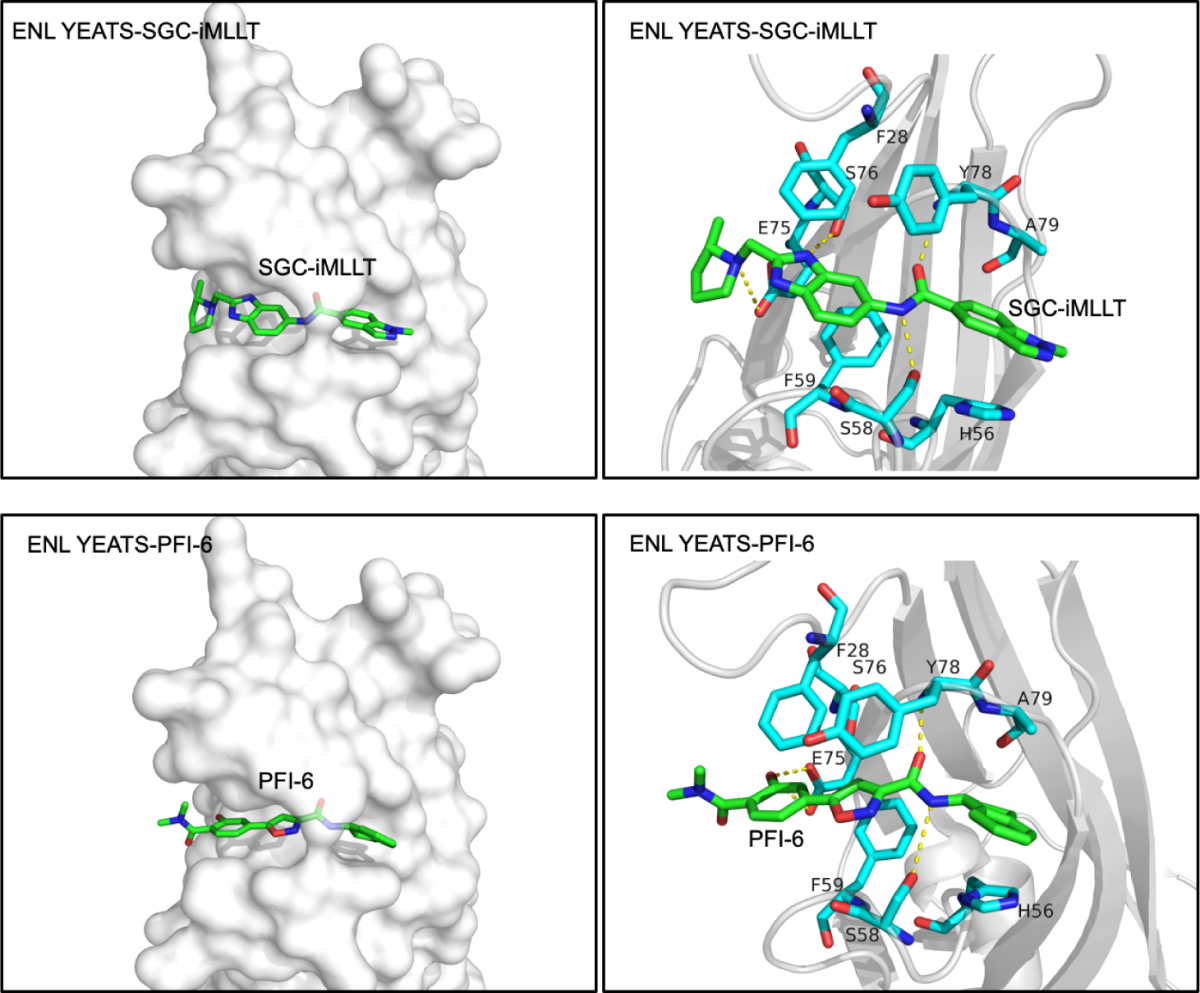
Structural insights into inhibition of the ENL YEATS domain by small molecules. (top panels) Crystal structure of the complex between the ENL YEATS domain and the chemical probe SGC-iMLLT (PDB: 6HT1). (bottom panels) Crystal structure of the complex between the ENL YEATS domain and the inhibitor PFI-6 (PDB: 8PJ7). Inhibitors are shown as green sticks. The ENL YEATS domain residues involved in the interactions (cyan sticks) are labeled. Yellow dashed lines represent hydrogen bonds. These structures illustrate distinct chemical strategies for targeting the YEATS domain and provide a structural framework for the rational design of selective inhibitors.

Subsequent biological studies demonstrated that TDI-11055 displaces ENL from chromatin, disrupts transcriptional elongation, and downregulates oncogenic gene expression, leading to cell differentiation. Importantly, TDI-11055 inhibited disease progression in xenograft models of MLL-rearranged and NPM1-mutated AML.^54^ To improve cellular activity and metabolic stability, other chemotypes such as piperazine-urea derivatives were explored. Among these, compound 1 emerged as a lead, showing low micromolar activity against ENL YEATS (IC_50_ = 3.7 μM, *K*_d_ = 5.5 μM) and moderate selectivity over AF9 (IC_50_ = 7.8 μM) with minimal or no activity against YEATS2 and GAS41.^55^ Although these compounds lacked aromatic stacking interactions with F28 and F59, they can potentially be engaged with distinctive binding pockets near loop 4 and thus present new avenues for scaffold hybridization. In parallel, Michael and coworkers employed high-throughput screening and SuFEx-based medicinal chemistry to develop SR-0813, a potent ENL YEATS inhibitor derived from an amido-imidazopyridine scaffold.^56^ SR-0813 bound ENL with a 30 nM affinity and selectively impaired the growth of ENL-dependent leukemia cells. Mechanistically, it evicted ENL from chromatin, suppressed leukemogenic transcription programs, and downregulated proto-oncogenes. However, its poor metabolic stability in mouse liver microsomes (*t*_1/2_ = 9.3 min) limited its in vivo potential.^57^

The methylisoxazole-carboxamide scaffold emerged as another promising chemical scaffold for targeting the YEATS domain. SAR optimization ultimately led to the development of PFI-6, which exhibited strong binding to ENL (IC_50_ = 140 nM) and AF9 (IC_50_ = 160 nM) with minimal activity against other YEATS family members. Crystal structures of the respective complexes confirmed that PFI-6 mimics acyllysine interactions within the YEATS binding pocket, validating its rational design (Figure 7). In cellular assays, PFI-6 showed robust targeting of AF9 in the NanoLuciferase Bioluminescent Resonance Energy Transfer (NanoBRET) assay, with an IC_50_ of 0.76 μM. While PFI-6 displays favorable physicochemical properties, high selectivity, and low cytotoxicity, making it a valuable chemical probe for studying ENL/AF9 biology, its limited antiproliferative activity and absence of in vivo pharmacokinetic data restrict its therapeutic potential.^58^

Recently, Liu and coworkers performed an AlphaScreen-based high-throughput screening (HTS) on a >66 000-compound library targeting the ENL YEATS domain and identified a novel class of amido-triazolopyridine inhibitors. Rational design introducing a salt-bridge interaction with E26 yielded compounds with <100 nM IC_50_ and *K*_d_ values and high selectivity over AF9.^59^ A NanoBRET assay was used to further guide the prioritization of inhibitor 13, which demonstrated strong cellular potency, metabolic stability, and antiproliferative effects in MLL-fusion leukemia cells. In AML xenografts, ∼45% tumor growth inhibition was achieved without notable toxicity at a 400 mg/kg dose. Inspired by the conversion of SGC-iMLLT to TDI-11055, we further optimized inhibitor 13 by replacing its benzyl group with a 1H-pyrrolo­[3,2-*c*]­pyridine and generated SR-C-107­(R). This modification improved hydrogen bonding contacts and introduced a potential salt-bridge interaction between the azetidine nitrogen and Glu75. SR-C-107­(R) showed potent anti-AML activity in both cell-based assays (CC_50_ = 1.25 μM in MOLM-13, 0.81 μM in MV4–11) and in vivo, underscoring its potential as a next-generation therapeutic agent for AML.^60^

Other chemical probes with different scaffolds have also been explored. sCGT990, a small-molecule inhibitor targeting the YEATS domains, was identified in an acellular thermal shift assay (CETSA). sCGT990 exhibited binding affinities of 14 μM and 62 μM toward the YEATS domains of ENL and AF9, respectively, and a minimal binding affinity (*K*_d_ > 150 μM) toward the YEATS domain of GAS41. sCGT990 belongs to a distinct chemotype compared to that of SGC-iMLLT.^61^ A spiro ent-clerodane homodimer with a rare 6/6/6/6/6-fused pentacyclic scaffold, isolated from , showed a potent inhibition of the YEATS domain of ENL (IC_50_ = 7.3 μM and *K*_d_ = 5.0 μM) (Figure 6).^62^ The Chaikuad group showed that oncogenic T1 and T3 ENL mutants have binding affinities comparable to those of wild-type ENL and remain susceptible to inhibition by acetyllysine mimetic compounds^63^ (Figure 6).

### PROTACs

Despite the advances in the development of peptide and small-molecule inhibitors targeting the ENL and AF9 YEATS domains, challenges such as functional redundancy and limited durability of target suppression remain. To address these issues and achieve more sustained target inhibition, attention has been turned to the development of proteolysis-targeting chimeras (PROTACs). By co-opting the ubiquitin-proteasome system, PROTACs enable selective degradation of YEATS domain-containing proteins, thereby offering a novel modality for therapeutic intervention that goes beyond simple occupancy of the binding site. To date, three distinct scaffolds, SR-0813, SGC-iMLLT, and PFI-6, have been utilized to generate PROTAC molecules capable of degrading ENL.^57,64,65^ These degraders, designed with either CRBN or VHL E3 ligase recruiters, suppress transcription of ENL target genes and demonstrate efficacy in mouse models of acute leukemia. SR-1114 (Figure 8), the first PROTAC derived from SR-0813, incorporates a thalidomide moiety (CRBN binder) linked via a polyethylene glycol spacer. In MV4-11 cells, SR-1114 induces rapid, CRBN-dependent ENL degradation with a DC_50_of 150 nM. However, ENL is rapidly resynthesized within 24 h after compound withdrawal, limiting its sustained activity and therapeutic potential. Cpd-1 (Figure 8), designed based on the SGC-iMLLT scaffold, is similarly conjugated to a CRBN ligand. It selectively degrades ENL without affecting its paralogue AF9 or other SEC components in MV4-11 leukemia cells. Notably, in vivo administration of Cpd-1 (30 mg/kg, i.p., daily) resulted in a significant tumor growth inhibition and prolonged survival in mouse models.^64^ More recently, MS41 (Figure 8), a potent and selective VHL-recruiting PROTAC derived from PFI-6, has demonstrated robust ENL degradation and inhibition of ENL-dependent leukemia cell proliferation. MS41 disrupts the chromatin occupancy of ENL-associated transcriptional machinery, represses oncogenic gene expression, and activates differentiation programs. Importantly, MS41 exhibits minimal toxicity and limited effects on normal hematopoiesis while markedly reducing leukemia progression in xenograft models.^65^

**Figure 8:**
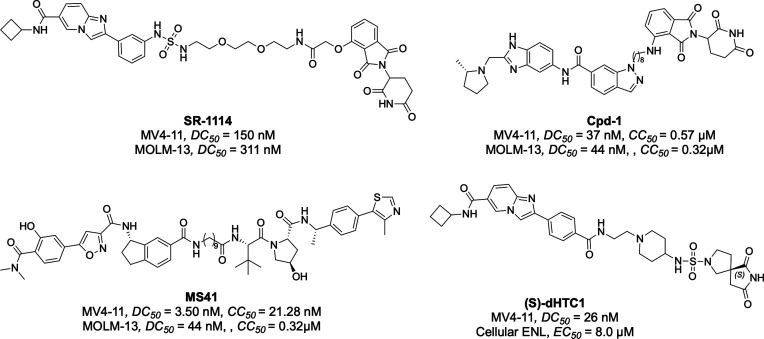
Representative PROTACs designed for the YEATS domains of ENL and AF9.

The Michael group used a high-throughput approach to discover chemical inducers of proximity (CIPs) based on sulfur­(VI) fluoride exchange (SuFEx) click chemistry. This method enabled the identification of a racemic compound, dHTC1. Further studies showed that the (S)-enantiomer, (S)-dHTC1 (Figure 8), but not its (R)-form, drives potent and selective degradation of ENL.^66^ Unlike traditional CRBN-reliant molecular glues or PROTACs, in this degradation pathway, the degrader bound to CRBN with high affinity only after forming the ENL:dHTC1 complex, as evidenced by the relatively weak binding affinity in CRBN FP assays (IC_50_ = 12 μM without ENL), which improved significantly (IC_50_ = 93 nM) in the presence of the ENL YEATS domain. Cellular studies confirmed that (S)-dHTC1 engages ENL with an EC_50_ of 8.0 μM and achieved a DC_50_ of 26 nM in MV4:11 cells. Furthermore, downregulation of ENL target genes, including HOXA10 and MYC, supports their targeted activity.

## GAS41 and YEATS2 YEATS Domains

Compound 5 was identified as a promising inhibitor of the GAS41 YEATS domain through an NMR-based fragment screening.^67^ Its ability to disrupt the interaction between the YEATS domain and histone H3K23crK27cr peptides was confirmed in Fluorescence Polarization (FP) and AlphaScreen assays, yielding IC_50_ values of 12 and 1.2 μM, respectively. These values were comparable to those obtained with an H3K27ac peptide (IC_50_ = 243 μM in FP and 24 μM in AlphaScreen assays). Structure-based optimization of compound 5 led to the development of more potent analogues (Figure 9). Compounds 12 showed markedly improved inhibitory activities, with IC_50_ values of 4.2 μM (FP assay) and 602 nM (AlphaScreen assay), respectively. Stereochemical analysis indicated a preference for the R-configuration of the proline moiety at the chiral center for optimal interaction with the GAS41 YEATS domain. Cocrystallization of compound 5 (Figure 10) with the GAS41 YEATS domain confirmed insertion of the inhibitor into the acyllysine-binding pocket and revealed critical interactions involving the thiazole and pyrrolidine rings. To enhance binding through a bivalent interaction mechanism, compounds 5 and 12 were dimerized to generate compounds 18 and 19. These dimers exhibited enhanced potency in disrupting GAS41 binding to diacylated histone peptides. Notably, compound 19 induced dimerization of the GAS41 protein and displayed superior binding affinity relative to its monomeric precursor. In cellular assays, compound 18 effectively disrupted the GAS41-histone H3 interaction in HEK293T cells, with an EC_50_ of ∼6 μM. Treatment with compound 19 stabilized endogenous GAS41 in H1299 cells, as confirmed by the Cellular Thermal Shift Assay (CETSA), whereas transcriptomic analysis revealed that compound 19 downregulated selected GAS41 target genes, indicating functional engagement with the YEATS domain and suppression of GAS41-mediated transcriptional activity. Compound 19 also appreciably inhibited the proliferation of multiple NSCLC cell lines in a GAS41-dependent manner.

**Figure 9:**
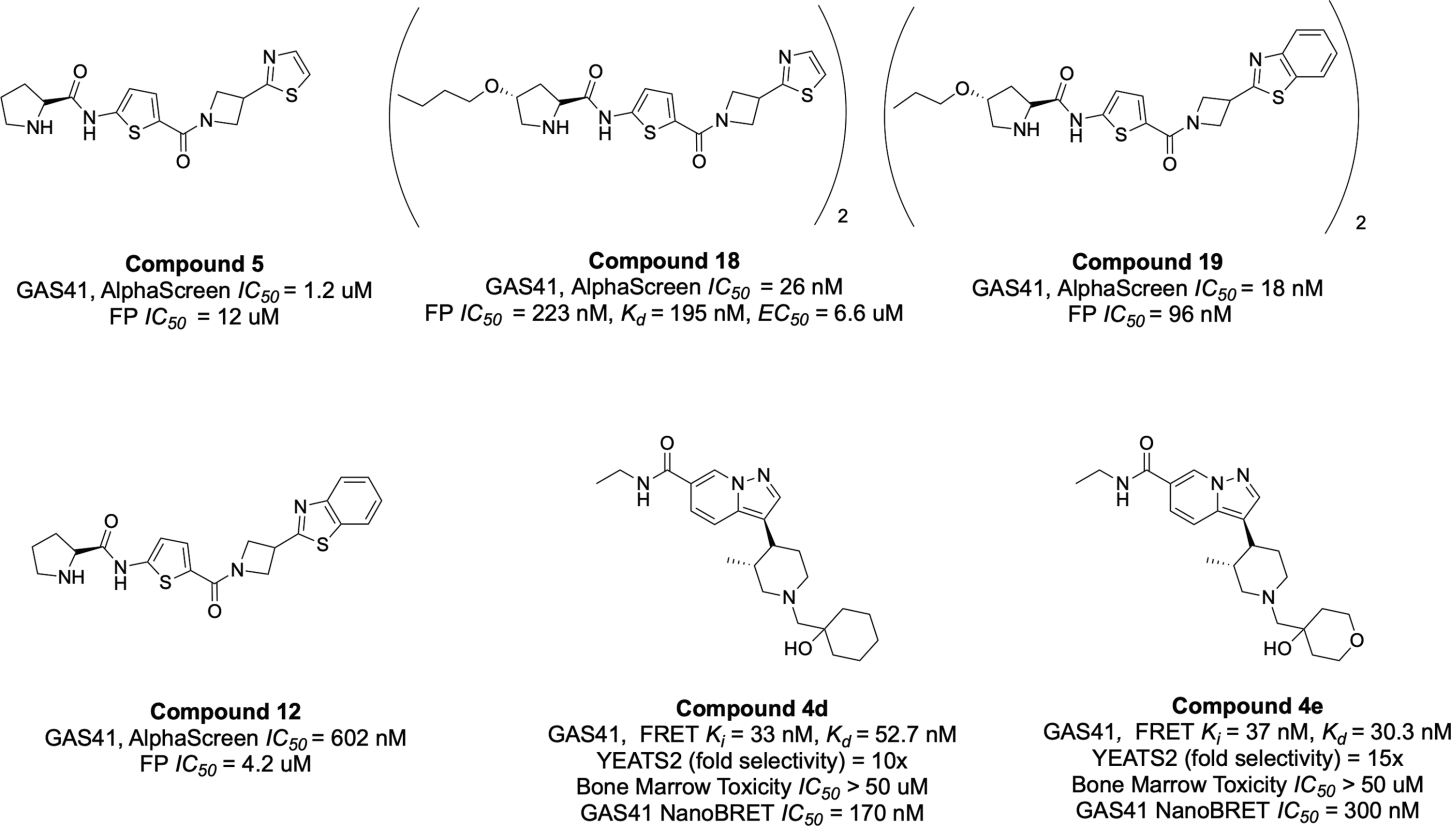
Representative small-molecule inhibitors and probes for the YEATS domains of GAS41.

High-affinity monovalent ligands targeting the GAS41 YEATS domain have also been successfully developed, originating from a benzimidazolone scaffold that was identified through virtual screening.^68^ Optimization of this hit led to the discovery of a potent pyrazolopyridine series with high selectivity for GAS41. Compound 4d, cocrystallized with the GAS41 YEATS domain, was shown to occupy the acetyllysine-binding site of the protein (Figure 10). Furthermore, compound 4d and 4e demonstrated strong inhibitory activity, with*K*_*i*_ of 33 nM and 37 nM, respectively. They also showed a strong binding affinity with a *K*_d_ of 52.7 nM and 30.2 nM determined by ITC and an IC_50_ of 170 nM and 300 nM measured in a NanoBRET assay. However, within this series, several synthesized compounds also showed activity against YEATS2. Given the high structural similarity between the YEATS domains of YEATS2 and GAS41, achieving absolute selectivity of small-molecule inhibitors for GAS41 remains a significant challenge.

**Figure 10:**
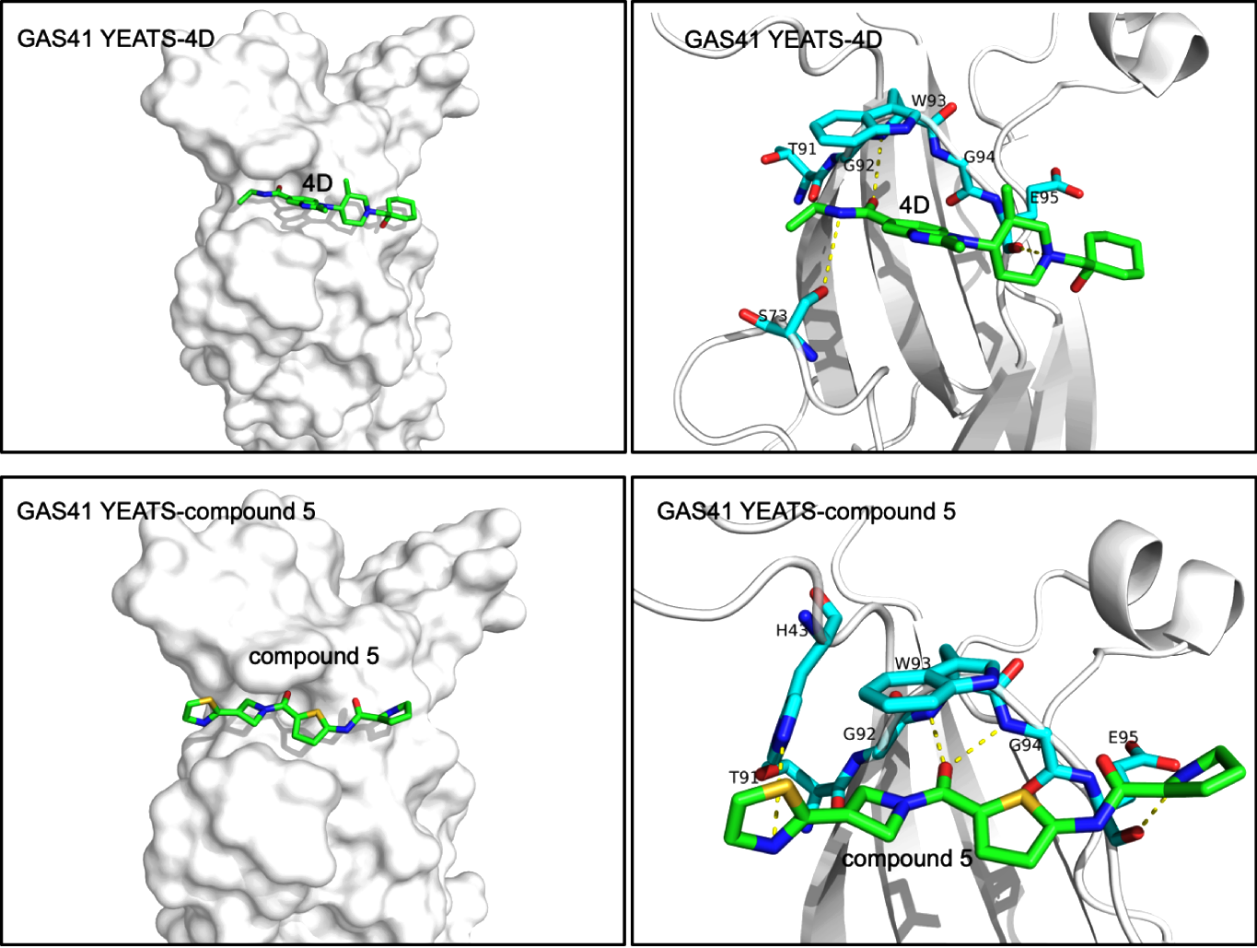
Structures of the GAS41 YEATS domain in complex with small-molecule inhibitors. (top panels) Crystal structure of the complex between the GAS41 YEATS domain and compound 4D (PDB: 8DKB). (bottom panels) Crystal structure of the complex between the GAS41 YEATS domain and compound 5 (PDB: 7JFY). Inhibitors are shown as green sticks. The GAS41 YEATS domain residues involved in the interactions (cyan sticks) are labeled. Yellow dashed lines represent hydrogen bonds.

## Outlook

Over the past decade, YEATS domains have emerged from mere structural scaffolds to critical epigenetic readers whose dysregulation drives human disease. Structural and functional studies of ENL, AF9, YEATS2, and GAS41 have revealed how each domain interprets diverse acyllysine modifications, such as crotonylation, acetylation, and even bulkier PTMs to fine-tune chromatin dynamics. Parallel advances in chemical biology have yielded a versatile toolkit of peptides, small molecules, and PROTACs that probe or disrupt YEATS-mediated transcription. Together, these insights lay the groundwork for the development of potential next-generation therapeutics while also highlighting persistent challenges.

First, achieving selectivity remains a major hurdle. ENL and AF9 share highly similar binding pockets, making most early inhibitors nonspecific. Recent breakthroughs-cyclized peptides and genetically encoded library selections-demonstrate that peripheral interactions outside the canonical cage can confer remarkable specificity. Extending this approach to YEATS2 and GAS41, and leveraging emerging allosteric or covalent strategies, could yield truly selective probes and drug leads. Chemical diversity also needs to be expanded beyond benzimidazoles and triazolopyridines: covalent warheads, macrocycles, and hybrid peptidomimetics may better fit to the shallow, open-ended YEATS ligand-binding channel and improve pharmacokinetics.

Second, in vivo efficacy hinges on overcoming poor metabolic stability and cell permeability. While PROTACs offer a powerful means to degrade ENL/AF9 and deliver sustained biological effects, current degraders suffer from the rapid resynthesis of target proteins or limited tissue distribution. Optimization of linker chemistry, alternative E3 ligases, and novel degrader modalities (e.g., molecular glues) could enhance potency, selectivity, and half-life in preclinical models.

Finally, future translational success will depend on the biological context and rational combination strategies. YEATS domains regulate inflammatory responses, neuronal gene expression, and DNA repair pathways. Systematic mapping of YEATS domain occupancy across cell types will uncover context-specific vulnerabilities. Combining YEATS inhibitors with other epigenetic agents, such as HDAC or bromodomain inhibitors, DOT1L antagonists, or immune modulators, may unlock synergistic antitumor effects. Advances in cryo-EM and AI-driven design are poised to accelerate the discovery of novel binding modes and allosteric pockets. As we deepen our understanding of the “acyllysine code,” the precise targeting of YEATS readers holds the promise of not only elucidating epigenetic mechanisms but also ushering in a new era of targeted, mechanism-based therapeutics.
